# TUB and ZNF532 Promote the Atoh1-Mediated Hair Cell Regeneration in Mouse Cochleae

**DOI:** 10.3389/fncel.2021.759223

**Published:** 2021-11-08

**Authors:** Zhenhang Xu, Vikrant Rai, Jian Zuo

**Affiliations:** ^1^Department of Biomedical Sciences, Creighton University School of Medicine, Omaha, NE, United States; ^2^Department of Otolaryngology, Xiangya Hospital, Central South University, Changsha, China

**Keywords:** hearing loss, hair cell, regeneration, transcription factors, atoh1

## Abstract

Hair cell (HC) regeneration is a promising therapy for permanent sensorineural hearing loss caused by HC loss in mammals. Atoh1 has been shown to convert supporting cells (SCs) to HCs in neonatal cochleae; its combinations with other factors can improve the efficiency of HC regeneration. To identify additional transcription factors for efficient Atoh1-mediated HC regeneration, here we optimized the electroporation procedure for explant culture of neonatal mouse organs of Corti and tested multiple transcription factors, Six2, Ikzf2, Lbh, Arid3b, Hmg20 a, Tub, Sall1, and Znf532, for their potential to promote Atoh1-mediated conversion of SCs to HCs. These transcription factors are expressed highly in HCs but differentially compared to the converted HCs based on previous studies, and are also potential co-reprograming factors for Atoh1-mediated SC-to-HC conversion by literature review. P0.5 cochlear explants were electroporated with these transcription factors alone or jointly with Atoh1. We found that Sox2+ progenitors concentrated within the lateral greater epithelial ridge (GER) can be electroporated efficiently with minimal HC damage. Atoh1 ectopic expression promoted HC regeneration in Sox2+ lateral GER cells. Transcription factors Tub and Znf532, but not the other six tested, promoted the HC regeneration mediated by Atoh1, consistent with previous studies that Isl1 promotes Atoh1-mediated HC conversion*ex vivo* and *in vivo* and that both Tub and Znf532 are downstream targets of Isl1. Thus, our studies revealed an optimized electroporation method that can transfect the Sox2+ lateral GER cells efficiently with minimal damage to the endogenous HCs. Our results also demonstrate the importance of the Isl1/Tub/Znf532 pathway in promoting Atoh1-mediated HC regeneration.

## Introduction

Hearing loss is one of the most common impairments affecting approximately 1.57 billion people worldwide, and there are 403.3 million people who still have moderate or severe hearing loss after adjusting for hearing aid use (Haile et al., 2021). Hair cells (HCs) in the inner ear play an essential role in converting mechanical movement to neural signals for hearing and balance (Youm and Li, 2018). HC death is the main pathology for hearing impairment and loss of HCs is one of the major causes of permanent sensorineural hearing loss in mammals. Non-mammalian vertebrates such as zebrafish, birds, and frogs still retain the capacity for the spontaneous regeneration of HCs. However, HCs in mature mammalian cochleae don’t regenerate spontaneously after damage (Zheng and Zuo, 2017).

HC regeneration is regarded as an ideal solution for complete hearing repair. In non-mammalian vertebrates, replacement of auditory and vestibular HCs occurs over a long period after birth. Supporting cells (SCs) are the source of newly generated HCs; direct transdifferentiation and mitotic regeneration are two main mechanisms of spontaneous HC regeneration (Cox et al., 2014). The basic helix-loop-helix (BHLH) transcription factor (TF) Atoh1 is a crucial player for HC development and regeneration (Liu et al., 2012; Whitfield, 2015; Lee et al., 2017). Compared to ectopic expression of Atoh1 alone, studies have shown that co-activation of Atoh1 together with other transcription factors (Pou4f3, Gfi1, Gata3, Nmyc, etc.) induces more SCs trans-differentiate to HC-like cells, and the newly generated cells are more mature in neonatal and mature murine cochleae (Liu et al., 2012; Walters et al., 2017; Chen et al., 2021), indicating the significance of co-activation of multiple factors relating to the HC regeneration.

We previously did the single-cell RNA sequencing (scRNA-seq) profiles of converted HCs (cHCs) and endogenous outer HCs (OHCs) after ectopically expressing Atoh1 in the Deiters’ cells (DCs) and Pilliar cells (PCs) at P14. scRNA seq revealed 16 transcription factors that were significantly differentially expressed in the endogenous HCs than in the cHCs. Among which Isl1 had been demonstrated to be a co-reprogramming factor that promotes Atoh1-mediated HC regeneration both ex-vivo and *in vivo* (Yamashita et al., 2018). Several of these 16 transcription factors including Six2, Ikzf2, Lbh, Arid3b, Hmg20a, Tub, Sall1, and Znf532 have been shown the potential for promoting the Atoh1-mediated regeneration, while Lbh, Ikzf2, and Six2 are the top three highly enriched transcription factors in adult OHCs compared to inner HCs (IHCs) with a fold difference of more than two (Li et al., 2016). Six2 may participate in complex formation with Eya1 and Sox2 for initial Atoh1 activation (Zhang et al., 2017), and can also interact with Eya1 to regulate the expansion of the nephron progenitors during nephrogenesis (Xu et al., 2014). Ikzf2 is a key regulator for the development and fate determination of OHCs (Chessum et al., 2018; Sun et al., 2021). Lbh is necessary for the maintenance of stereocilia bundles and the survival of cochlear HCs (Liu et al., 2020). Arid3b involves in embryonic development, regulation of cell lineage, and cell cycle control (Kortschak et al., 2000). Hmg20a plays a role in neuronal differentiation and can preserve neuronal integrity (Lorenzo et al., 2021). A mutation of Tub causes loss of both tectorial membrane-attachment crowns (TM-ACs) and horizontal top connectors (HTCs), leading to severe hearing loss (Han et al., 2020). Sall1 mutation is responsible for Towners-Brocks syndrome (TBS) by displaying high-frequency sensorineural hearing loss in humans (Yang et al., 2021). Znf532 is a potential common target of Stat3 and Wnt/Pcp pathways (Savino et al., 2019). The expression of Tub and Znf532 was also regulated by Isl1 (Liang et al., 2015); Isl1 is essential for the development of pacemaker cells and helps to prevent age-related and noise-induced hearing loss (Huang et al., 2013; Liang et al., 2015).

Ectopic HC regeneration occurred in different subtypes of SCs, including greater epithelial ridge (GER), inner border cells (IBCs), inner phalangeal cells (IPhCs), pillar cells (PCs), Deiters’ cells (DCs), Hensen’s cells, and lesser epithelial ridge (LER) in the organ of Corti after ectopic overexpression of Atoh1 (Liu et al., 2012, 2021; Richardson and Atkinson, 2015; Walters et al., 2017; Yamashita et al., 2018). Sox2, an HMG domain transcription factor, is a marker for the neurosensory domain of the otic placode and is critical for the initiation of progenitor’s transdifferentiation to HCs by interacting with the 3’ enhancer of Atoh1, the expression of Sox2 decreases to almost absent at P7 in the HCs (Kempfle et al., 2016; Cheng et al., 2019). GER cells are a group of transient cells that will be replaced with inner sulcus cells during the postnatal maturation of the organ of Corti; at neonatal ages, the lateral GER are abundant of Sox2+ cells which have more plasticity and capacity for transdifferentiation (Kubota et al., 2021), regenerated HCs were acquired by ectopically overexpressing Atoh1 in GER cells in cultured cochlear explants through electroporation (Zheng and Gao, 2000). All of these indicate that the lateral GER cells can serve as progenitor sources for HC regeneration at neonatal ages.

We hypothesized that Six2, Ikzf2, Lbh, Arid3b, Hmg20a, Tub, and Sall1 which are differentially expressed between the endogenous HCs and the cHCs and that are highly expressed in the endogenous OHCs might serve as cooperative factors for Atoh1 mediated HC regeneration. To test our hypothesis, we optimized the cochlear explant electroporation procedure and electroporated these transcription factors alone or together with Atoh1 to see if anyone of these transcription factors or the combination could induce more regenerated HCs in the GER cells.

## Materials and Methods

### Animals

All inbred FVB males and females were purchased from Jackson Laboratory (Stock Number 001800). Breeding pairs were set up to get P0.5 pups for the electroporation. All animals were kept at the animal facility of Creighton University following standard care. The animals were kept under a 12–12 h light-dark cycle at 72–75°F with a normal diet ad libitum. The protocol for all experiments was approved by the Institutional Animal Care and Use Committee (IACUC) of Creighton University.

### Plasmid DNA Preparation

All plasmids were prepared in the lab with EndoFree Plasmid kits (Qiagen, cat#12391) following the manufacturer’s protocol. Briefly, the plasmid DNA transformation was done using HD5-alpha competent cells and incubated by shaking in an agar medium to develop antibiotic resistance. The bacterial culture was grown on agar plates and four different colonies were collected for batch culture. After culturing the bacterial particles overnight, particles were pelleted and plasmid preparation was done using a maxi-prep kit from Qiagen following manufacturers’ instructions. The yield of plasmid DNA diluted in HBSS buffer was estimated using Nanodrop 2000 from ThermoScientific. High quality of plasmid was essential for successful transfection. Plasmid solutions were stored at −20°C and diluted to 0.5 μg/μl in HBSS (without calcium, magnesium, and phenol red) before being used for electroporation.

### Tissue Cultures and Electroporation

The temporal bones were dissected out immediately after the sacrifice of the neonate and transferred into the iced HBSS (Thermo Scientific, cat#88284). Micro tweezers were used to remove the bony cochlear wall, Reissner’s membrane, and spiral ligament to expose the basilar membrane entwining around the modiolus. The isolated cochlea sensory epithelium was transferred to the center of the Millipore filter membrane (Millipore Sigma, PICM03050) using trimmed 200 μl tips. We used a similar electroMasudaporation method as reported by Masatsugu and made some optimizations (Masuda et al., 2012); we didn’t moisten the filter by adding HBSS, the epithelium was submerged in more than 20 μl HBSS on the filter to avoid dehydration. The epithelium was reset to make sure HCs were facing up, HBSS around the tissue was removed by pipette, and plasmids were added on top of the tissue. The concentration of each plasmid was kept at 0.5 μg/μl and the total volume of plasmids was kept at 6 μl for each electroporation to transfect 3 μg plasmid for each gene in total. The epithelium was electroporated using the electroporator (CUY21EDIT, NEPAGENE) and 2 rectangular pulses (12 V, 30 ms duration with an interval of 970 ms) were applied. The filter with explant was transferred to the 6-well culture plate, plasmid solution was removed, tissue was rinsed with pre-warmed Opti-MEM and left undisturbed in 1 ml Opti-MEM for 1 min. After removing all the Opti-MEM, 1.5 ml pre-warmed culture medium (DMEM/F12, 10% fetal bovine serum, 1% N-2 supplement, 2% B-27 supplement, 50 μg/ml ampicillin) was added to the well, and 200 ul culture media was added to the filter, the first drop of the media was added directly on top of the tissue so that the tissue wouldn’t float off. The explants were cultured in the incubator with 5% CO_2_ and 95% humidity at 37°C for several days, and the culture media was changed every 24 h.

### Immunofluorescence Analysis

Explants were collected 2, 3, 4, and 6 days after the electroporation and fixed in 4% Paraformaldehyde (cat#15710, Electron Microscopy Sciences) for 15 mins; the tissues were then blocked for 1 h in 0.2% Triton X-100 and 10% (v/v) heat-inactivated goat serum in PBS. Tissues were then incubated with the primary antibodies overnight at 4°C and corresponding secondary antibodies for 2 h at room temperature. The following primary antibodies were used: anti myosinVI (1:400, cat #25-6791, Proteus Bioscience), anti-GFP (1:400 cat # A10262 Invitrogen). All secondary antibodes were purchased from Invitrogen and used at a 1:800 dilution. DAPI (4′,6-diamidino-2-phenylindole) was used to counterstain the nucleus at 1:1,000 dilutions (Cat#D1306, Invitrogen).

### Quantification and Statistical Analyses

All images were taken by Zeiss LSM 700 confocal microscope to minimize the variation among samples. All slides were examined by confocal microscopy using the 10× lens to locate the apical-middle turn of the explant, then turn to 40× lens for taking the Z-stack images at a 1-μm interval. All original files were analyzed using the software ZEN (black edition). Transfected GER cells within 70 μm distance from the endogenous HCs were counted; we excluded PCs that were adjacent to the endogenous HCs and occasionally transfected. GFP+ cells were counted as transfected cells, the GFP+/MyoVI+ cells in the GER region were counted as converted HCs (cHCs), and GFP−/MyoVI+ cells were counted as endogenous HCs. Three explants were inspected per electroporation. All data analysis was performed using one-way ANOVA and the Student’s t-test with a Bonferroni correction in Graphpad Prism 9.0.

## Result

### Gene Delivery by Electroporation Is Efficient in Transfecting Sox2+ Lateral GER Cells

**Figure 1:**
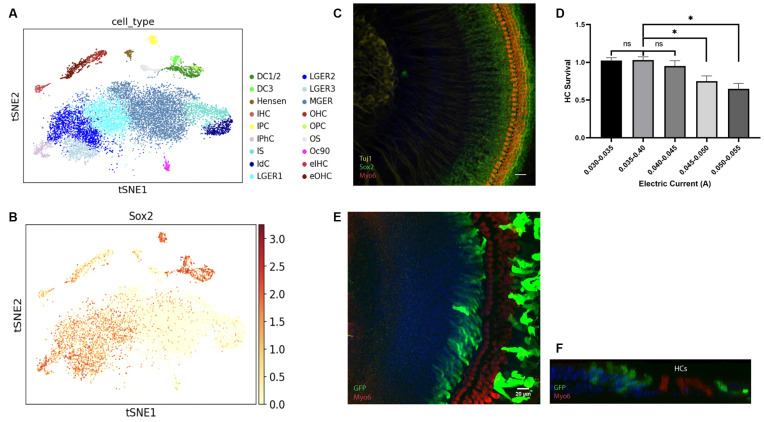
**(A–C)** Identification of Sox2+ cells in the cochlea from the neonatal mouse. **(A–B)** tSNE plot for ~14,000 cochlear cells collected from P1 mice. **(A)** Distinct cell types within the cochlea are labeled with different colors. **(B)** Feature plots showing the expression of Sox2 for the same cells in **(A)**. Expression is concentrated in the IPC, DC, lateral GER with a low expression level in HCs. **(C)** Localization of expression for Sox2 in P0.5 cochlear explants. HCs are labeled with Myosin6 and spiral ganglion neurons are labeled with Tuj1. **(D–F)** The high efficiency of gene delivery to the lateral GER is mediated by electroporation. **(D)** HC survival rate in explants applied with different electric currents. **(E)** The HCs are intact and barely electroporated, GFP+ cells indicated transfected cells located in the lateral GER and lesser epithelial ridge (LER). **(F)** A representative orthogonal image showing the transfected GFP+ epithelium cells. Scale bars: 20 μm. *P < 0.05 and ns, not significant by unpaired two-tailed Student’s *t*-test. Results are presented as the mean ± SEM.

HCs are developed from the Sox2+ progenitor cells in the prosensory domain at the embryonic stage (Kempfle et al., 2016). We first used Kolla et al’s scRNA-seq data from postnatal day 1 (P1) mice to validate the Sox2+ cell population at P1 (Kolla et al., 2020). As shown in Figures 1A,B generated using gEAR Portal, DCs, IPhCs, and lateral GER cells are all Sox2+ while some of the endogenous HCs also have low Sox2 levels (Walters et al., 2015; Orvis et al., 2021).

We then examined the Sox2+ progenitors in the GER at P0.5, within the organ of Corti. Sox2 is expressed in the SCs and lateral GER cells within 70 μm distance from the endogenous HCs and its expression level gradually decreases in the GER area away from the endogenous HCs (Figure 1C), which is similar to previous studies (Dabdoub et al., 2008; Kubota et al., 2021). As the lateral GER is abundant in Sox2+ progenitors, they are excellent target cells for electroporation for the HC regeneration study.

Electroporation-based gene delivery has been widely performed in different tissues (Kawabata et al., 2004; Voyer et al., 2018) as it is an efficient approach. The cochlear explant electroporation has been reported (Zheng and Gao, 2000; Driver and Kelley, 2010); here we further optimized the protocol to increase the stability and minimize the variability of the results. The plasmid encoding GFP driven by a constitutive (CMV) promoter was used for evaluating the location and efficiency of gene delivery by electroporation. The cochlear basilar membranes were dissected out from P0.5 FVB pups and transfected with the GFP plasmid. The cochlear explants were then cultured for 3 days and harvested for GFP staining to visualize the transfected cells. Myosin6 staining was used to detect the endogenous HC distribution in the explant. We found that even when the parameters were set up identically, the transfection efficiency and HC viability varied, and the viability of HCs decreased when the electric currents were higher than 0.045 amperes (A) (Figure 1D).

When electric currents were controlled within 0.030–0.045 A, strikingly, we found most of the transfected cells in cochlear explants were preferentially located in the Sox2+ lateral GER, and few endogenous cells were transfected (Figure 1E). All the transfected GFP+ GER cells were not Myo6+, which means none could transdifferentiate to Myo6+ cells spontaneously. Orthogonal images also showed that transfected cells in the GER were just medial to endogenous HCs and located on the surface of the epithelium (Figure 1F).

### Atoh1 Ectopic Expression Promoted HC Regeneration in Sox2+ GER Cells

**Figure 2:**
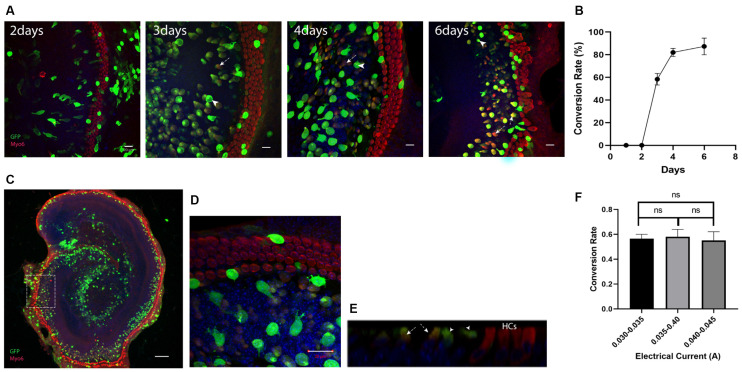
Ectopic expression of Atoh1 leads to the new Myosin6 expressing cells in lateral GER. **(A)** Morphological images of Atho1-eGFP plasmids transfected explants at 2, 3, 4, and 6 days after the electroporation. The image for 6th day is an enlarged image of the white square in panel **(C)**. GFP+ cells indicate the transfected cells and GFP+/Myo6+ cells in lateral GER indicate the Myo6 expressing cHCs after the ectopic expression of Atoh1. There were no cHCs 2 days after the electroporation, more cHCs were observed from day 3 to day 6 , and the transfected cells were gradually getting closer to endogenous HCs. **(B)** The percentage of GFP+/Myo6+ cells vs. all GFP+ cells in lateral GER (conversion rate) gradually increases from day 2 to day 10 after the electroporation. **(C)** Typical Atoh1-eGFP plasmid transfected cochlear explant showing most of the transfected cells were localized in the lateral GER, Scale bar = 100 μm. High-resolution image **(D)** and ortho image **(E)** showing the transfected epithelium cells. **(F)** The conversion rate of transfected cells with different electric currents. The arrows pointed to GFP single positive cells which are the transfected cells that failed to transdifferentiate, the arrowheads pointed to the GFP, Myosin6 double-positive cells which are the cHCs. Scale bar = 20 μm. ns, not significant by unpaired two-tailed Student’s *t*-test. Results are presented as the mean ± SEM.

Atoh1 has been reported as a key factor for HC regeneration at neonatal age (Zheng and Gao, 2000; Chen et al., 2013). To confirm the efficiency of regeneration using this electroporation method, cochlear explants were electroporated with the Atoh1-eGFP plasmid. The number and duration of electric pulses or other parameters applied to the tissue were identical for all experiments. Electric currents were restricted within 0.03–0.045 A. The GER cells labeled with anti-GFP antibodies were transfected cells, and the double labeling cells with anti-GFP and anti-Myo6 antibodies were regarded as cHCs (Figure 2).

To detect the gradual change of conversion rates after the electroporation, we performed immunostaining 2, 3, 4, and 6 days after the electroporation. At 2 days after the electroporation, none of the transfected cells expressed Myosin6, the cHCs appeared at 3 days after the electroporation. Just like the electroporation for eGFP plasmid, 6 days after the electroporation, most of the transfected cells were concentrated in the Sox2+ lateral GER region (Figure 2C). We then analyzed the transfected lateral GER cells within a 70 μm distance from the endogenous HCs for calculating the conversion rate. We could see the conversion rate gradually increased and cHCs migrated much closer to the endogenous HCs 6 days after the electroporation (Figures 2A,B). Three days after the electroporation, the conversion rate was around 60% and we chose 3 days after the electroporation as the time point for testing other TFs, because we could detect any increase or decrease of the conversion rate easily with additional factors. The conversion rate of the transfected cells had no statistical difference between different explants when the current was below 0.045A (Figure 2F).

### Tub and ZNF532 Promote the Conversion Mediated by Atoh1 in Lateral GER

**Figure 3:**
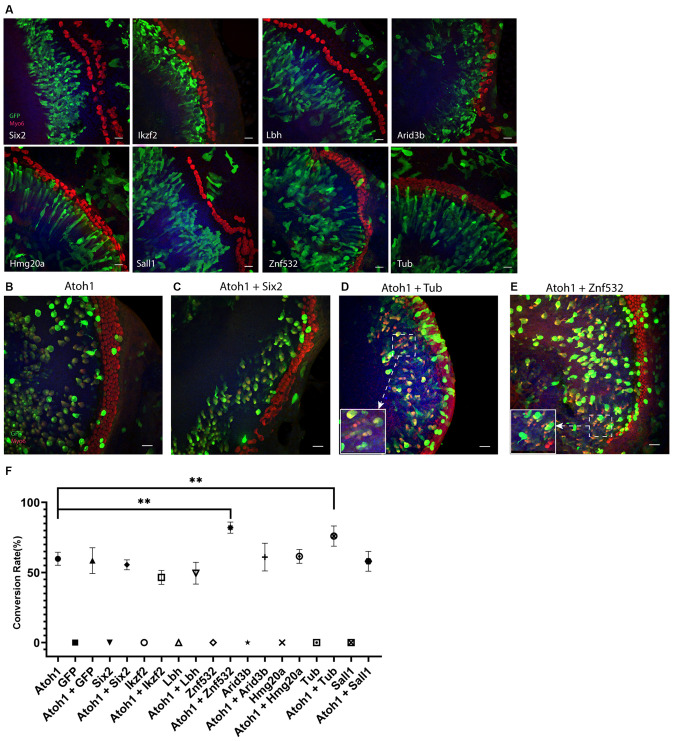
**(A)** Forced transcription factor expression alone in lateral GER cells didn’t lead to any conversion. **(B–E)** Ectopic expression of Tub or Znf532 promotes the Atoh1 mediated conversion in lateral GER. **(C)** A representative negative control showing Six2 failed to promote the Atoh1 mediated conversion. **(D,E)** The explants shown were transfected with combinations of Atoh1 + Tub and Atoh1 + Znf532. **(F)** Statistical data showed that there are significant increases in the conversion rate with Atoh1 + Znf532 and Atoh1 + Tub compared with Atoh1 alone. Scale bar= 20 μm. **P < 0.01 by unpaired two-tailed Student’s *t*-test. Results are presented as the mean ± SEM.

We tested multiple transcription factors including Six2, Ikzf2, Lbh, Arid3b, Hmg20a, Tub, and Sall1 which are expressed highly in the HCs and differentially compared to the cHCs (Yamashita et al., 2018) and are also potential co-reprograming factors for Atoh1-mediated SC-to-HC conversion by literature review. To test our hypothesis, we first electroporated the explants with these individual transcription factors alone; all plasmids used for the electroporation was 0.5 μg/μl, the total volume of plasmids for each gene was 6 μl. To get higher transfection efficiency and better explore the transdifferentiation capacity of each of these transcription factors, electric current was not restricted below 0.045 A and all explants were cultured for 4 days after the electroporation. The cochlear explants were similar to GFP plasmid transfected explants and none of the transfected GER cells were Myo6+. This suggests that none of these transcription factors alone have the capacity to convert Sox2+ GER cells to Myo6-expressing cHCs (Figure 3A).

Co-electroporations of Atoh1 with each of these transcription factors were performed where the concentration of the plasmid of either Atoh1-GFP or tested transcription factors was 0.5 μg/μl and 6 μl was used for the electroporation. The explants were imaged 3 days after the electroporation (Figures 3B–E). We analyzed all transfected lateral GER cells within a 70 μm distance from the endogenous HCs for all groups. The conversion rates of either different genes alone or different combinations are shown separately (Figure 3F). Atoh1 alone induced about 60% transfected cells to be Myosin6+ CHCs, co-electroporation of Atoh1 with Tub or Znf532 significantly increased the conversion rate in lateral GER whereas all other combinations of transcription factors with Atoh1 failed to change the conversion rate of Atoh1 alone.

## Discussion

HC regeneration is one of the ideal treatment strategies for hearing recovery. The co-manipulation of multiple transcription factors has been proved to be critical for efficient HC regeneration (Masuda et al., 2012; Walters et al., 2017; Chen et al., 2021), and more studies are needed to find more gene targets. Gene delivery methods for the inner ear cells include viral vector-based-transduction and non-viral vector-based transfection (Sacheli et al., 2013). Plasmid electroporation for cochlear explants has been developed and performed in embryonic and neonatal mice as it is convenient and efficient for gene delivery; however, there are still some barriers that limit its use. In our study, we optimized and improved the procedure in the following aspects. We minimized the time for the whole procedure; this requires the experimenter to have high anatomical skills. The tendency of the tissues to float off the incubation surface has been discussed previously (Parker et al., 2010). Instead of culturing cochlear explants on the polyornithine/laminin-coated glass coverslips, we utilized the Millipore filter membrane to mimic the *in vivo* environment for the organ of Corti. The cochlear tissue was placed on the membrane after being dissected out until the fixation. A small part of the filter membrane with explant was cut off with a micro scalpel and used for immunofluorescence analysis. We found the cochlear explants attached to the membrane very well and barely float off.

Given that Sox2+ progenitor cells at neonatal ages have high plasticity and capacity for transdifferentiation, studies have been done to isolate the Sox2+ cells from neonatal mice, and the isolated progenitor cells were either cultured to organoids or directly used for further study (Kempfle et al., 2016; Kubota et al., 2021). The utilization of unisolated *in situ* Sox2+ cells for regeneration research has additional advantages as the intact organ of Corti mimics the *in vivo* condition. As our purpose was to transfect the single layer of Sox2+ lateral GER cells located on the surface of the cochlear sensory epithelium, the electric current was controlled to below 0.045 A, a very low electric current level, by adjusting the distance of anode and cathode. We minimized the damage of electroporation and ensured that the transfected cells are almost all Sox2+ lateral GER cells. The expression of Myo6 takes less than 2 days after the initiation of Atoh1 during the development of organ of Corti (Driver et al., 2013). However, at 2 days after the electroporation, none of the transfected cells expressed Myosin6, which means the transdifferentiation *in vitro* takes more time compared to the normal developmental process.

The combinations of multiple transcription factors have been proved as necessary for fate determination and efficient HC regeneration. Isl1 is a transcription factor that can promote the Atoh1 mediated HC regeneration *ex vivo* (identical to our explant transfection assays reported here), and it has been further proven in a transgenic overexpression mouse model that Isl1 does promote Atoh1-mediated HC conversion *in vivo*, further validating our explant assays. Moreover, the overexpression of Isl1 in HCs can also help to prevent age-related and noise-induced hearing loss (Huang et al., 2013; Yamashita et al., 2018). Strikingly, the two transcription factors (Tub, Znf532) we identified here as cofactors for Atoh1 mediated conversion are also regulated by Isl1 (Mu et al., 2008; Liang et al., 2015). The Tub transcription factor is critical for the normal function of TM-ACs and HTCs. Stat3 signaling has been demonstrated to regulate the HC regeneration in zebrafish, and both Stat3 and Wnt/PCP pathways play important roles in mouse HC differentiation (Montcouquiol and Kelley, 2003; Liang et al., 2012; Waqas et al., 2016; Chen et al., 2017), interestingly, Znf532 is a potential common target of Stat3 and Wnt/Pcp pathways. All of these suggest that the interplay of Isl1, Tub, Znf532, Stat3, and Wnt/Pcp pathways play essential roles in HC regeneration and possibly other regenerative systems. As the newly identified transcription factors Tub/Znf532 are regulated by Isl1, further studies will be warranted to explore the function of the Isl1/Tub/Znf532 pathway during HC regeneration.

In conclusion, our studies reveal an optimized electroporation method that can transfect the Sox2+ lateral GER cells efficiently with minimal damage to the endogenous HCs. Our results also indicate the importance of the Isl1/Tub/Znf532 pathway in promoting the Atoh1-mediated HC regeneration.

## Data Availability Statement

The raw data supporting the conclusions of this article will be made available by the authors, without undue reservation.

## Ethics Statement

The animal study was reviewed and approved by Institutional Animal Care and Use Committee (IACUC) of Creighton University.

## Author Contributions

ZX, VR, and JZ conceived the experiments. ZX and VR performed experiments. ZX performed data analyses, prepared figures, and wrote the manuscript. All authors contributed to the article and approved the submitted version.

## Conflict of Interest

The authors declare that the research was conducted in the absence of any commercial or financial relationships that could be construed as a potential conflict of interest.

## Publisher’s Note

All claims expressed in this article are solely those of the authors and do not necessarily represent those of their affiliated organizations, or those of the publisher, the editors and the reviewers. Any product that may be evaluated in this article, or claim that may be made by its manufacturer, is not guaranteed or endorsed by the publisher.
